# Notes and Notices

**Published:** 1883-10

**Authors:** 


### NOTES AND NOTICES.

Erratum.—Dr. E. Rushmore, not Dr. Mills, is Chairman of Bureau of Materia Medica, I. H. A.

Locations in Nebraska.—Twenty-five good locations in Nebraska. Address Dr. G. E. Brown, Secretary, Nebraska State Homœopathic Society, Albion, Nebraska.

Homœopathy as a Science.—An excellent article on this subject will be found in *Popular Science Monthly* for October, from the pen of Dr. Edward Bayard.

Virchow.—The German physicians are very rigid in their adherence to the *Code.* Prof. Virchow has now fallen under their displeasure for thanking a druggist for some pills sent him and used with benefit. The enterprising druggist published this letter to advertise himself. The *faculty* attacked Virchow for recommending, as they allege, quack preparations. Virchow refuses to be "bull-dozed," gets mad, and resigns from the German Society of Physicians. He declares "that he can no longer belong to a Society that arrogates to itself the right of so arbitrary and offensive a criticism. It is to be hoped that a similar spirit of petty obtrusiveness, such as is shown in this instance, does not now, and never will, prevail among the medical faculty."

A Chair of Homœopathy.—A homœopathic physician at Vienna has left a large sum of money for the establishment of a homœopathic chair in the Vienna University, but the Austrian Government has declined to accept it.—*Chicago Medical Journal*, Sept., 1883.

To Stop Hiccough.—Dr. W. E. Shaw, of Cincinnati, Ohio, writes: "Place the tips of the fingers of both hands in position of complete supination against the abdominal muscles, at the lower and outer junctions of the epigastric with hypochondriac regions. With the finger-tips in this position, firm and very gradual pressure was to be made backward and upward against the diaphragm. This pressure should be continued for some little time after the diaphragm has ceased its spasmodic contractions, when the fingers should be very gradually withdrawn."—*Medical Record.*

Dr. Bristowe on Sanitary Science.—Dr. John Syer Bristowe, President of the Society of Medical Officers of Health, confessedly one of the most sagacious observers and logical thinkers of the day, writes as follows: "If we look to the remarkable influence which *simple variations of temperature and peculiarities of season* exert on the mortuary returns, in respect both of the number of deaths and the character of the fatal diseases, and compare therewith the comparatively small effect on the death rate of even one of the most fatal of the zymotic diseases, or with the insignificant influence of death from enteric fever, diphtheria, and other affections, *over which sanitary science is supposed to exert a specially valuable influence*, we can scarcely avoid seeing that, on similar grounds, the deaths saved directly by the sanitary labors on which we are engaged must, under any circumstances, be so few annually as to produce *no distinct and ummistakable effect on the mortuary rate.*"

This sincere and ingenuous avowal of opinion by one not only distinguished as an author and teacher, but who, in his exalted official position, had every opportunity to scrutinize all facts regarding outbreaks of typhoid fever, scarlet fever, and diphtheria, as bearing upon the question of their prevention, may well be commended to the serious consideration of those who, for years past, have positively promised the extinction of these diseases, under certain impossible conditions, yet with no other result than that just presented by the above-named conscientious and eminent authority in medical science, Dr. George Hamilton.—*Medical Record.*

Rings.—The Tweed Ring is long since dispersed, the Star Route Ring is broken, but the Ring that controls the Institute is still strong!

